# Automatic real-time monitoring and assessment of tremor parameters in the upper limb from orientation data

**DOI:** 10.3389/fnins.2014.00221

**Published:** 2014-07-24

**Authors:** Stefan Lambrecht, Juan A. Gallego, Eduardo Rocon, Jose L. Pons

**Affiliations:** Neurorehabilitation group, Cajal Institute, Spanish National Research Council (CSIC)Madrid, Spain

**Keywords:** tremor, MEMS, sensor location, context awareness, real-time estimation

## Abstract

Upper limb tremor is the most prevalent movement disorder and, unfortunately, it is not effectively managed in a large proportion of the patients. Neuroprostheses that stimulate the sensorimotor pathways are one of the most promising alternatives although they are still under development. To enrich the interpretation of data recorded during long-term tremor monitoring and to increase the intelligence of tremor suppression neuroprostheses we need to be aware of the context. Context awareness is a major challenge for neuroprostheses and would allow these devices to react more quickly and appropriately to the changing demands of the user and/or task. Traditionally kinematic features are used to extract context information, with most recently the use of joint angles as highly potential features. In this paper we present two algorithms that enable the robust extraction of joint angle and related features to enable long-term continuous monitoring of tremor with context awareness. First, we describe a novel relative sensor placement identification technique based on orientation data. We focus on relative rather than absolute sensor location, because in many medical applications magnetic and inertial measurement units (MIMU) are used in a chain stretching over adjacent segments, or are always placed on a fixed set of locations. Subsequently we demonstrate how tremor parameters can be extracted from orientation data using an adaptive estimation algorithm. Relative sensor location was detected with an accuracy of 94.12% for the 4 MIMU configuration, and 100% for the 3 MIMU configurations. Kinematic tracking error values with an average deviation of 8% demonstrate our ability to estimate tremor from orientation data. The methods presented in this study constitute an important step toward more user-friendly and context-aware neuroprostheses for tremor suppression and monitoring.

## Introduction

Pathological tremor encompasses all types of tremors that impair motor performance (e.g., essential tremor and parkinsonian tremor; McAuley and Marsden, 2000), and is the most common movement disorder (Wenning et al., 2005). Sixty five percent (Elble and Koller, 1990) of tremor patients report serious difficulties in the performance of their activities of daily living (ADL) (McAuley, 2000; E Rocon, 2004; Wenning et al., 2005). Furthermore, patients suffering from pathological tremor experience functional disability to the extent that it can lead to social isolation. In this article we refer to pathological tremor as tremor.

Recently new tremor treatment strategies, based on mechanical loading, have been proposed in addition to the existing therapies. These novel strategies are deemed necessary given the low success rate and side effects induced by both drugs and neurosurgery in some types of patients; in 25% of patients tremor is not managed satisfactorily (Rocon et al., 2007b). Tremor suppression through mechanical loading is based on the principle that tremor amplitude can be modified by altering limb impedance through the application of force or by adding mass (Adelstein, 1981; Prochazka et al., 1992; Rocon et al., 2007a). For example, Rocon et al. demonstrated for the first time that a wearable robot that applied force to the upper limb segments could effectively attenuate upper limb tremors (Rocon et al., 2007a). Other studies have shown that it is possible to attenuate the tremor using the human muscle tissue as actuators, through functional electrical stimulation (Javidan et al., 1992; Popović Maneski et al., 2011; Gallego et al., 2013; Bó et al., 2014). Functional electrical stimulation neuroprostheses avoid a heavier and more obtrusive rigid structure (Gallego et al., 2011).

To avoid constant actuation and the reduction of tremor without functional improvement, total movement must be separated into voluntary and tremulous movement (Rocon et al., 2007a). This is typically performed using adaptive algorithms (see e.g., Gallego et al., 2010; Bo et al., 2011). Tremor suppression devices subsequently intervene only when tremor coincides with voluntary movement. Unlike wearable robots, where most sensors are embedded in the device, neuroprostheses depend on additional sensors. Both MEMS accelerometers and gyroscopes are used to monitor tremor (Grimaldi et al., 2008; Elble, 2009). For example, the neuroprosthesis presented in Gallego et al. (2013) implemented microelectromechanical (MEMS) gyroscopes for measuring tremor. Accelerometers constitute the most popular approach. They however measure linear acceleration, in contrast to human motion which is considered as rotations about joints. Furthermore, there is no accepted model to separate gravity from voluntary motion in the accelerometer data (Veltink et al., 1996; Sabatini, 2011). Gyroscopes measure angular velocity and therefore provide a more direct representation of human movement. Gyroscopes are thus more adequate than accelerometers to extract tremor characteristics from motion data; however they do suffer from a low-frequency bias resulting in an integration drift. This bias does not affect the estimation of tremor, but is inherently present in the voluntary movement component of the signal. The presence of this integration drift inhibits the accurate extraction of joint angles from gyroscope data over longer periods of time (>10 s) (Woodman, 2007).

MEMS limitations are often addressed by sensor fusion. The most common approach is to correct the gyroscope data with accelerometers and magnetometers (Foxlin, 2002). Currently the most popular fusion method is Kalman filtering. In magnetic and inertial measurement units (MIMU) the accelerometer and magnetometer data is used to reset the bias of the gyroscopes in quasi-static periods or after filtering the accelerometer and magnetometer data (Roetenberg et al., 2005; Sabatini, 2011). We refer to Sabatini (2011) for more information on sensor fusion and the use of MEMS in human motion analysis. MIMUs thus allow us to obtain orientation data by small, relatively unobtrusive sensors that can be incorporated into a garment (see e.g., Gallego and Rocon, 2011).

To enrich the processing of long-term tremor monitoring and to increase the intelligence of the neuroprosthesis we need to be aware of the context. Context is defined as “any information that can be used to characterize the situation of an entity” (Dey, 2001) and can refer to a situation (being in a meeting, driving a car) or an activity the patient is performing. Context awareness is a major challenge for neuroprosthesis and would allow these devices to react more quickly and appropriately to the changing demands of the user and/or task. This would also permit to monitor the evolution of the therapy provided by the neuroprosthesis, and the evolution of the patient's condition. Kinematic features are traditionally used to increase context awareness. A substantial body of literature supports the use of body-worn sensors for context and ADL classification (Farringdon et al., 1999; Kunze et al., 2005; Kunze and Lukowicz, 2007; Korel, 2010). Their availability and low cost have made accelerometers the most wide spread sensor modality used to extract kinematic features. Recent advances in MIMUs and their incorporation into the latest generation of consumer electronics however are rendering robust orientation data easily available. To accurately obtain joint angles over time, we need a robust measurement of the orientation or position of each segment over time. Joint angles and features derived from joint angles have recently demonstrated their potential (Ofli et al., 2012) and are gaining in popularity with the advent of more wearable and affordable motion capture equipment. In this paper we propose a first step toward increased context awareness for neuroprostheses for tremor management.

In order to be able to extract joint angles it is vital to know where the sensors are placed on the body. Automated sensor location identification facilitates the donning and doffing of patients by medical doctors for instrumented analysis or by the patients themselves, for use of tele-rehabilitation devices or neuroprostheses at home. Little or no research has been done to identify sensors location on the body. So far only one study Kunze and Lukowicz (2007) has looked at sensor placement identification in tasks other than walking. A limitation is that a 6 min window was needed to achieve 85% accuracy for 4 sensor locations spread across the body. The majority of ADLs are shorter in duration, moreover is it not recommendable for our application that the patient endures such a lengthy calibration period. Other studies started from the hypothesis that the patient would be walking, and predominantly focused on sensor placement on the lower limbs (Kunze et al., 2005; Kunze and Lukowicz, 2007; Vahdatpour et al., 2011; Weenk et al., 2013). All previous work has been based on accelerometer data. Weenk et al. were the first to also introduce gyroscopes in an attempt to make their classifier more location invariant. Assuming that the body consists of rigid body segments angular velocity is invariant to location on the segment. Weenk et al. furthermore used characteristics of the walking cycle to achieve orientation invariance. They took advantage of the specific characteristics of walking and made assumptions related to the quality of movement execution. The participant was assumed to be walking in a straight line, the direction of which was subsequently used to transform from local to global sensor orientation. No upper limb task exists that has such stable and repetitive characteristics as walking. Movement disorders moreover severely disrupt task execution in such a way that dominant direction is corrupted by involuntary movement and thus advocate for more easily applicable localization methods.

Here we present a novel method to automatically identify relative sensor location on the upper limb. Our approach is based on an upper limb task, and relies on the observation that movement and tremor are more pronounced distally. We demonstrate that features extracted from the movement we selected can be used to identify relative sensor location based on orientation data. We focus on relative sensor location rather than pure sensor location, because in many medical applications MIMUs are used in a chain stretching over adjacent segments (e.g., Analysis of kinematics, tele-rehabilitation applications), or are always placed on a fixed set of locations (e.g., gait segmentation). In the particular application with a neuroprothesis, this algorithm facilitates the re-instrumentation (placing of the sensors) after cleansing the fabric. The main contribution of this sensor location algorithm in monitoring applications is that it ensures correct and accurate measurements without the need for prior (technical) knowledge. Further, this identification algorithm can be combined by standard MIMU-to-body calibration routines to obtain anatomical joint angles. Subsequently we demonstrate for the first time how tremor can be extracted from orientation data. Therefore, using orientation data we are able to identify sensor location, estimate tremor and derive context information from the same dataset, thus reducing bandwidth requirements.

## Materials and methods

### Subjects

A group of 6 patients (3 male, 3 female; 63.2 ± 11.8 years) affected by essential tremor was recruited for this study. The patients were diagnosed by the neurological personnel of the Hospital 12 de Octubre as definite essential tremor, according to the criteria described in Deuschl et al. (1998). Tremor severity was 30.2 ± 13.0 (ranging from 10 to 48) according to the Fahn-Tolosa-Marin rating scale (Fahn et al., 1998). Patients continued taking their regular medications at the time of the recordings. Informed consent was obtained from all patients prior to starting data collection. Approval for this study was obtained through the Ethics committee of the Hospital 12 de Octubre, granting its accordance to the Declaration of Helsinki.

#### Protocol

Patients were asked to perform a finger-to-nose test in repetitive manner while seated. The patient was asked to alternatively touch the nose and knee with the tip of his/her right index finger. Contact with nose and knee had to be maintained for a few seconds during each repetition; the total trial duration was 30 s. Two trials of each patient were analyzed, with a single trial consisting of 3 finger-to-nose cycles. Finger-to-nose is typically used in neurological examinations to activate kinetic tremor (Deuschl et al., 1998). Essential tremor is predominantly manifested during task execution. Finger-to-nose furthermore shares the main kinematic pattern with a multitude of ADLs related to the upper limb such as drinking, eating, and personal hygiene.

### Instrumentation

**Figure 1:**
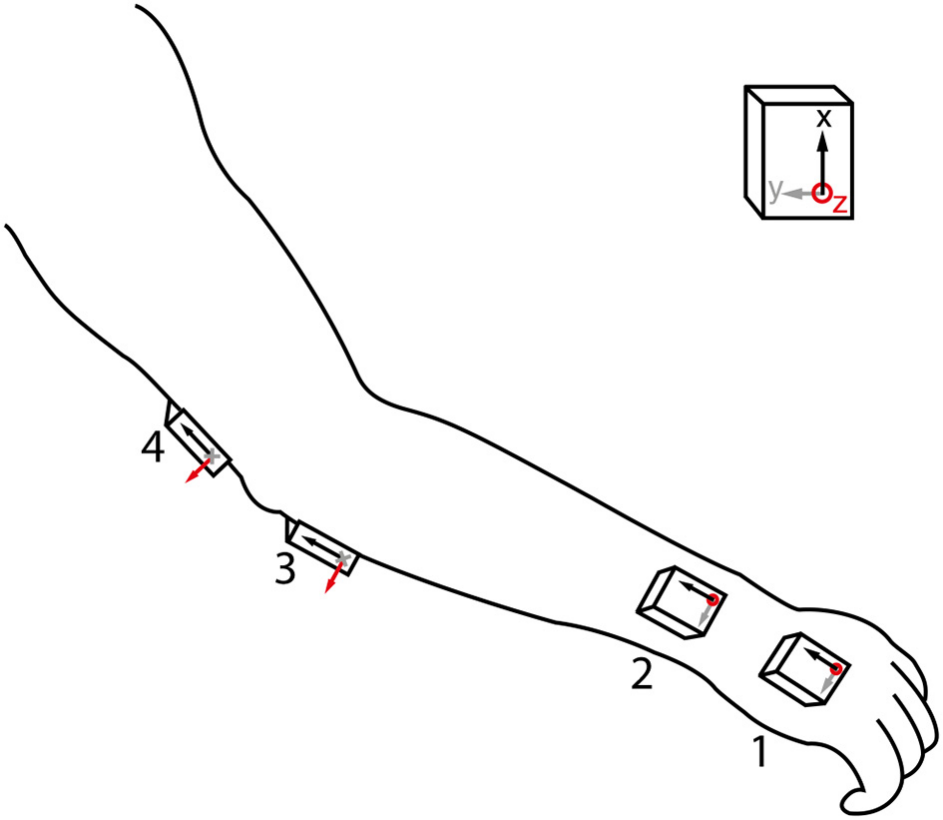
**MIMU sensor placement**. Axes of the sensors were aligned with the main joint axes in setup, and placement sites with little soft tissue were selected. The 4 MIMU configurations consists of sensors 1-2-3-4, the 3 MIMU configurations are 1-2-4 and 1-3-4.

We used 4 MIMUs (Tech MCS, Technaid S.L., Madrid, Spain) comprising tri-axial accelerometers, gyroscopes, and magnetometers to measure upper limb kinematics (sampling rate: 100 Hz). They are particularly suited for the estimation of tremor due to their low weight (40 g) and small size (11 × 26 × 36 mm). The sensors were attached with double sided hypo-allergenic tape to the hand, distal forearm, proximal forearm, and humerus (Figure 1). Orientation was calculated by the onboard extended Kalman fusion (EKF) algorithm. Proper alignment between sensor axes and anatomical axes was ensured upon placing the MIMUs. Fixation on soft tissues was avoided to prevent low pass filtering of the motion signal and to eliminate the influence of undesired soft tissue oscillations (Tong and Granat, 1999). In addition to the configuration shown in Figure 1, based on the current design of the neuroprosthesis, we also tested a subset more commonly used in biomechanics with only one sensor per segment (hand, forearm, and humerus).

### Data analysis

#### Sensor location identification

We have chosen features related to angular velocity and thus need to decompose the orientation data into angular velocity. In a three-dimensional scenario, as is the case with orientation data, we cannot obtain angular velocity by direct differentiation of attitude angles. The non-vectorial nature of finite angular displacements nullifies this assumption. We therefore use the Poisson equation to extract the angular velocity $\left[\dot{\theta }\right]$ (Zatsiorsky, 1998):

$$[\dot{\theta }]=[\dot{\text{R}}]{\left[\text{R}\right]}^{-1}$$

Where $\left[\dot{\text{R}}\right]$ represents the rate of change of the direction cosines and [R]^−1^ corresponds to the body attitude. This equation has been used to identify the instantaneous helical axis (Veldpaus et al., 1988).

Based on pilot work on a mechanical mockup and healthy subjects (Lambrecht and Pons, 2014) we selected 18 candidate features (Table 1). In an attempt to make our features orientation invariant, we rectified the sensor data and combined information from all axes (|x|,|y|,|z|). Our approach is further based on the observation that the kinematic chain has an additive effect regarding movement of individual segments, i.e., movement of proximal segments is (partly) represented in more distal segments. To an extent this pattern is also noticeable in tremor, being more manifest at distal than at proximal segments.

**Table 1:** **Selected features for classification of sensor location**.

**Displacement**	**Velocity**	**Acceleration**
**1. ∑(Total distance (|x|, |y|, |z|))**	**5. ∑(Max(|x|, |y|, |z|))**	9. ∑(Max(|x|,|y|,|z|)
**2. ∑(RMS(|x|, |y|, |z|))**	**6. ∑(RMS(|x|, |y|, |z|))**	10. ∑((RMS(|x|,|y|,|z|))
**3**. $\sqrt{\left(RMS\left(|x|,|y|,|z|,\right)\right)}$	**7**. $\sqrt{\left(RMS\left(|x|,|y|,|z|,\right)\right)}$	11. $\sqrt{\left(\text{RMS}\left(|\text{x}|,|\text{y}|,|\text{z}|,\right)\right)}$
**4. ∑(var(|x|, |y|, |z|))**	**8. ∑(var(|x|, |y|, |z|)**	12. ∑(var(|x|,|y|,|z|))
**13. ∑(eigenvalues of covariance matrix(|x|,|y|,|z|)), displacement**		
**15. ∑(eigenvalues of covariance matrix(|x|,|y|,|z|)), velocity**		
17. ∑(eigenvalues of covariance matrix(|x|,|y|,|z|)), acceleration		
14. ∑(principal component coefficients), displacement		
16. ∑(principal component coefficients), velocity		
18. ∑(principal component coefficients), acceleration		

To achieve a classification that is robust across various levels of tremor and sensor orientations each feature is based on the rectified values across all axes of each MIMU. The root mean square (RMS) and variance (var) values are calculated over the full trial duration. The values marked in bold are those resulting in high classification performance.

A total of three sensor configurations were adopted, the current neuroprosthesis (NP) setup as shown in Figure 1 and two configurations each with one MIMU per segment. The latter two differed in the location where the second sensor is placed, being respectively distal and proximal on the forearm. All features were used as ranked values to enhance robustness of the classifiers across intensities of tremor (nearly absent to severe). These classifiers were: random forest, decision tree, and ranking.

Random forest classification generates an ensemble of “bagged” decision trees with random feature and sample selection, each such combination is also referred to as a “bag.” In each bag a decision tree is trained on a bootstrap or subsample of the initial data set. The benefit of a random forest over decision tree is that the ensemble of trees can lead to a better result than the best individual tree. We calculate the accuracy of the prediction of the random forest as out-of-bag error, reflecting the accuracy in identifying sensor location for data not used in a specific bag, The random forest was programmed using the treebagger algorithm in Matlab, selecting 4 leaves and 100 trees.

The decision tree, one of the most successful techniques for supervised classification learning, is more intuitive than the random forest and computationally less demanding. However, one has to be careful to not overtrain the tree. Overtraining occurs when the classifier reaches a maximum accuracy for the training data used, but performs poorer on new data than a classifier that was not overfitted to the training sample. To identify and avoid overtraining we compute both the resubstitution and 10-fold cross-validation error. The resubstitution error reflects the accuracy of the classifier on the training data. In the case of decision trees, resubstitution error will keep decreasing upon adding nodes to the tree. The cross-validation error represents the misclassification occurring on new data, not used for training. We optimize the combined cost of resubstitution error and cross-validation error and added a 1 standard deviation window to this value to ensure avoiding an over-fitted sub-optimum. We used the classregtree function in Matlab to compute the decision trees.

Ranking can be considered a form of classification, in particular when applied to chains of sensors. The most important benefits of ranking are that no training is needed and that the configuration of sensors can thus be modified without penalty. Ranking furthermore has a negligible computational cost. The advantage is that the chain of sensors can be shortened or elongated, and slid up or down without the need for retraining or changing between classifiers. The only requirement is that the configuration is known beforehand. The features listed in Table 1 were individually sorted in descending values; the thus obtained vector was then compared to the reference vector. The reference for all of the above methods is the fixed order in which the sensors were placed on the subjects, starting distally (MIMU 1 placed on the hand, see Figure 1).

#### Tremor estimation

The orientation data was passed by the same protocol as used by the classification, after which the angular velocity estimate was upsampled to 1 kHz. To obtain joint motion we subtracted the angular velocity data from sensors proximal and distal to the respective joint (Rocon et al., 2006). We focused our analysis on the wrist joint because tremor is more present and disabling further down the kinematic chain (Belda-Lois et al., 2004).

To estimate wrist tremor from the raw movement we used the algorithm presented in Gallego et al. (2010). This algorithm assumes that tremulous and voluntary movement can be separated by frequency distribution. The frequency of voluntary movement during the execution of ADL is between 0 and 2 Hz (Riviere, 1996), with mean around 1 Hz (Mann et al., 1989). Tremor frequency range between 3 and 12 Hz (Deuschl et al., 1998) By estimating the voluntary movement with a g-h filter (Brookner, 1998), and subtracting it from the raw movement data we obtained an estimate of the tremulous movement. The parameter of the g-h filter was set by optimization with a genetic algorithm over all trials, minimizing the total cost over all patients and trials. Since our intention was to reduce the tremor component in the signal, we set bounds at 0.8 and 1. Lower values would likely result in a too high tremor to voluntary motion ratio in the signal. Selection of the initial data was dome randomly with uniform spacing, using a population size of 100. We further applied a crossover rate of 80% with 2 elitist survivors in mutation, and a roulette method for natural selection. The fitness function minimized the kinematic tracking error (KTE) (see below). The parameters thus obtained for the gyroscope and orientation data are respectively 0.9952 and 0.9958.

We compared our results to the online and offline methods based on gyroscope data presented in Gallego et al. (2010). The offline method is considered a gold standard or ideal reference method (Rocon et al., 2006), but cannot be implemented in the control of a neuroprosthetic. The online gyroscope method is used as a reference to compare our results to a practical alternative for real-time tremor estimation. The offline method consists of filtering gyroscope data with a recursive low pass filter (fc = 2 Hz).

The performance of the orientation based tremor estimation was assessed through the KTE. KTE consists of two components that together evaluate the smoothness, response time and execution time of a tracking algorithm relative to a reference method (Rocon, 2006).

$$KTE=\sqrt{\varphi^{2}\left|b\right|+\sigma^{2}\left|b\right|}$$

Where φ^2^ |*b*| represents the mean of the absolute estimation error (*b* = |*y*_*k*_ − *x*_*k*+1,*k*_), and represents how fast the algorithm is capable of reacting when velocity changes. The offline gyroscope estimation, x_*k* + 1,*k*_, is used as reference in the error calculations. The second component σ^2^ |*b*| is the variance of the absolute estimation error and gages the smoothness of the estimated variable.

## Results

### Sensor location identification

Table 2 summarizes the results, percentage of MIMUs identified correctly, of the various classifiers for all configurations tested. Performance was unaffected by altering the forearm sensor location from distal to proximal, therefor we report average values in Table 2. All classifiers achieved a perfect score for the setup when only 1 MIMU was attached to each segment (left column). In the 4 MIMU configuration, with 2 MIMUs attached to the forearm, a decrease in performance was observed (right column Table 2). The fact that gyroscope data (gray) provides the best classification performance indicates that angular velocity is sufficient to identify relative sensor location. Similar results were achieved using the orientation data (black). The poorer result obtained with the decision tree using orientation data was likely due to an overly conservative correction in the cross-validation. Only taking the resubstitution error into account, the accuracy achieved by the decision trees was 0.975. The results from ranking further support this hypothesis.

**Table 2:** **Performance of the classifiers on each of the different sensor configurations**.

	**3MIMUs**	**4MIMUs**
**Random forest**	1	0.9560
	1	0.9412
**Decision tree**	1	0.9559
	1	0.8824
**Ranking**	1	0.9412
	1	0.9412

The values in gray refer to the gyroscope data, those in black to the orientation data. A score of 1 corresponds with perfect classification, 0 corresponds with incorrect classification of all sensors.

**Figure 2:**
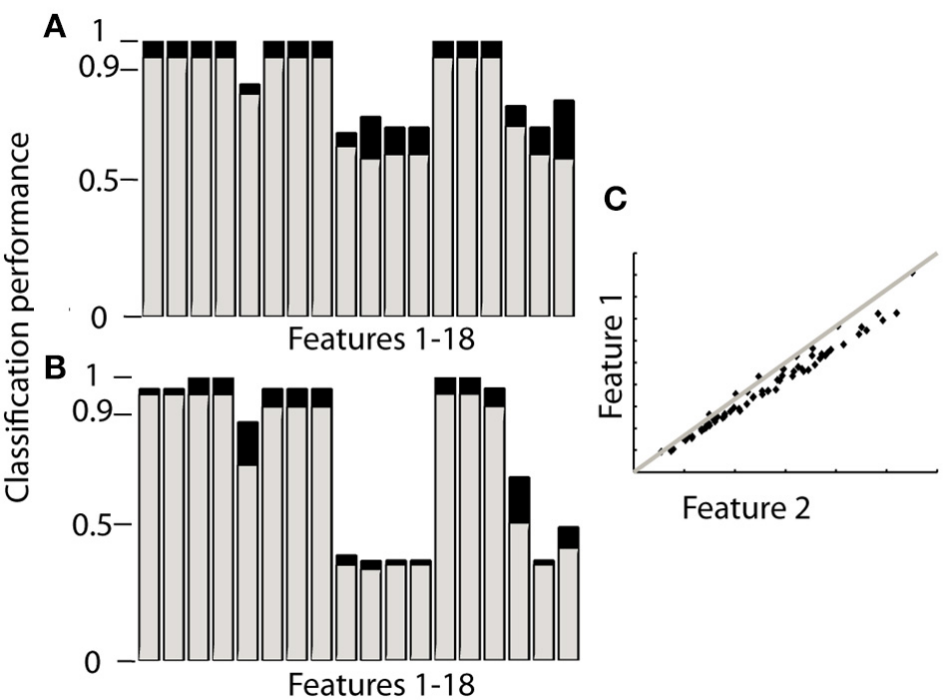
**Performance of each feature to identify sensor location based on ranking**. The gray bars in **(A,B)** correspond to the 4MIMU configurations, the black bars to the configurations with 3 MIMUs. The orientation data is presented in **(A)**, and the gyroscope data in **(B)**. In **(C)** the redundancy of the features is demonstrated by contrasting features 1 and 2, using orientation data both resulting in high scores in **(A,B)**. **(C)** Is representative of the redundancy among the 10 features highlighted in Table 1.

Ranking proved to be the best option since it does not need training and reached similar levels of accuracy as the other classifiers. When using orientation data 10 features each provided the maximum accuracy reported in Table 2 (Figure 2A), these features are marked in bold in Table 1 and depicted in Figure 2. Figure 2 furthermore shows that only 4 features achieve this level of accuracy when using gyroscope data (Figure 2B). Features proved to perform equally well across subjects and highly redundant amongst each other (Figure 2C). Either of the features marked in bold in Table 1 thus resulted in a similar classification performance.

### Tremor estimation

**Figure 3:**
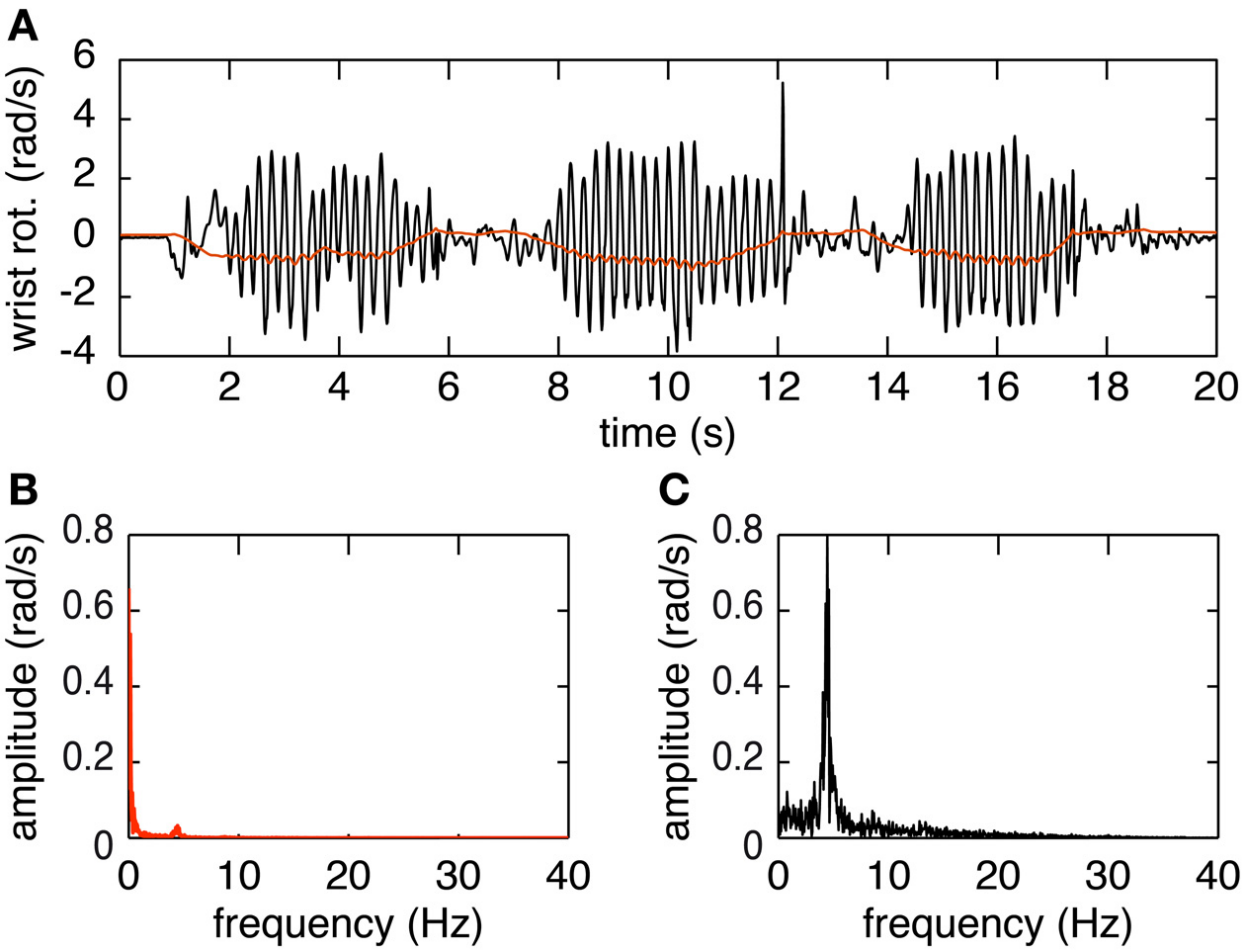
**Example of the decomposition of orientation data during a finger-to-nose test. (A)** Shows the orientation data decomposed using the Euler method (in red) and the raw movement (in black). **(B)** Shows the amplitude spectrum of the orientation data decomposed using Euler. **(C)** Shows the amplitude spectrum of the orientation data decomposed using the proposed decomposition method.

The plots in Figure 3 provide an overview of the decomposition process. In Figure 3A the joint angle obtained by Euler decomposition is shown in red. This signal predominantly represents the voluntary motion, due to the filtering process done by the EKF used for orientation estimation. Tremor frequency is nonetheless preserved in the orientation (Figure 3B). The first peak, at 0–2 Hz, represents the voluntary movement whereas the second, much smaller, peak at ~5 Hz corresponds to tremulous movement. The decomposed signal using the method presented in this paper is depicted in black in Figure 3A. The frequency spectrum of this signal indicates that decomposing in this form allows us to extract the tremor characteristics but with a loss of the voluntary signal. This however is not an issue since the voluntary movement is present, with little to no signs of tremor, in the orientation data and can easily be accessed directly extracting the Euler angles from the rotation matrix.

**Figure 4:**
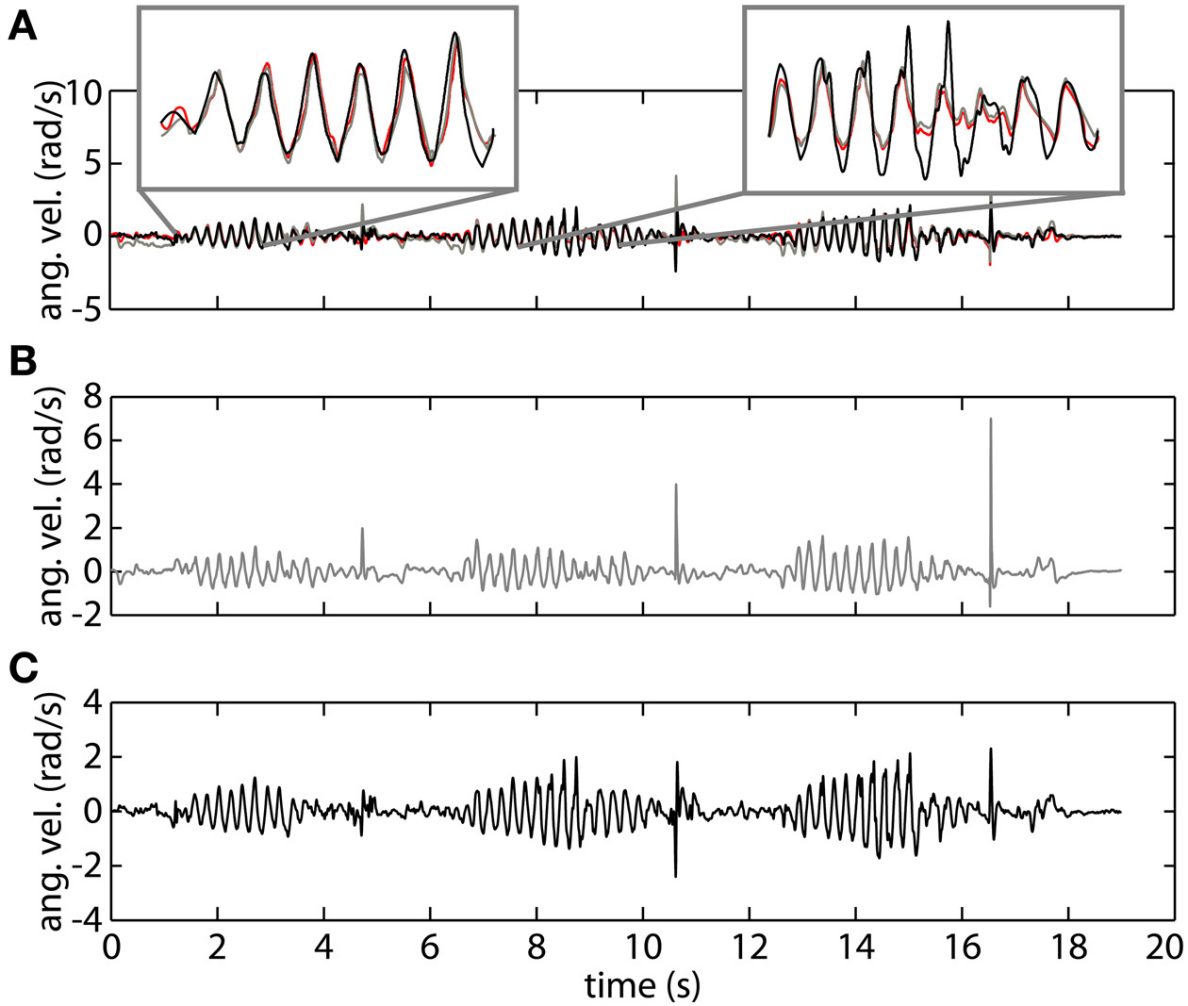
**An example of tremor estimation using the proposed online method based on orientation data (black), an online method based on gyroscope data (gray), and an offline reference method based on gyroscope data (red). (A)** Shows all the signals together. **(B)** Represents tremor estimated using the online gyroscope method. **(C)** Shows the tremor estimate obtained using the method proposed in this paper.

In Figure 4 we show a representative trial using both gyroscope references, online and offline, as well as the proposed method using orientation data. The top plot demonstrates the high correspondence of the proposed method with both the online gyroscope method and an offline gold standard method. The first highlight showcases the strength of the orientation based method, following both the online and offline gyroscope tremor estimates closely in amplitude and in frequency. The second highlight places attention to a limitation of the presented method. It appears that upon changes in velocity the orientation method is slow in adjusting; the orientation based method in black deviates from both the gold standard in red and the online gyroscope method. We assume that this is due to the intrinsic characteristics of the onboard EKF of the MIMUs used. The EKF parameters are set to track voluntary human movement, characterized by a lower frequency than the tremor we are tracking.

**Figure 5:**
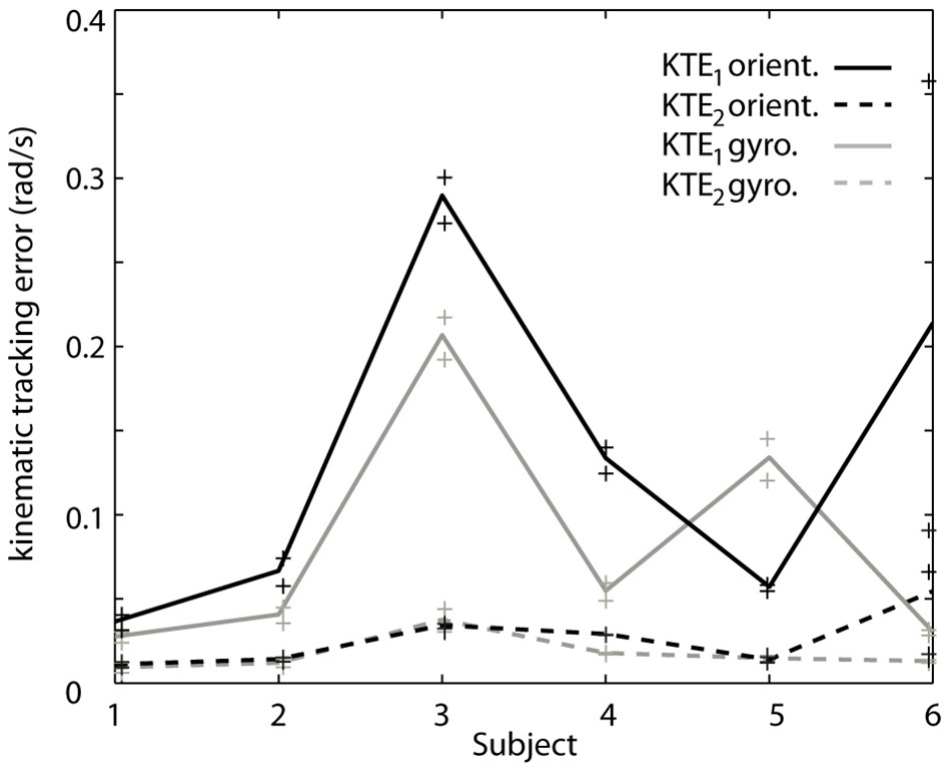
**Contribution of each of the components of the KTE representing relative performance of the gyroscope method (gray) and the orientation based method (black) relative to an offline reference method based on gyroscope data**. The KTE_1_ (mean of the absolute tracking error) is represented by the continuous lines, whereas the KTE_2_ (variance of the absolute tracking error) is plotted in dotted lines. The data plotted in the curves is the average across trials for each patient, minimum and maximum performance is represented by “+” for each patient.

To verify the hypothesis that the EKF is a limiting factor when changes in velocity occur, we analyze both components of the KTE separately (Figure 5). It is clear that the differences are predominantly present in the first component (KTE_1_; the mean of the absolute estimation error), and thus related to the response time of the algorithm. We believe that adapting the EKF could increase the performance of the presented method. KTE values of both online methods, comparing each method to the gold standard, did not differ more than 8% with respect to the value of the orientation based KTE. Mean KTE of the online gyroscope method was 0.2963 ± 0.1146 (min: 0.11750; max: 0.5137), and for the method proposed in this paper 0.3704 ± 0.1548 (min: 0.2034; max: 0.6730). A more direct analysis was not possible in the current study since the EKF used was embedded in the MIMU and acted as a “black box.” Future studies should further investigate what the effect of the fusion algorithm is on errors in tremor estimation from orientation data. The current results, although preliminary due to the small sample size and high inter-patient variability, appear to indicate that orientation data is suitable for NP control. Orientation data are likely best combined with an impedance modulation control strategy for the NP (Gallego and Rocon, 2011). Impedance control is less reliant on highly precise data than noise canceling approach. In impedance control, the viscosity and stiffness of the joints are increased to generate a low pass filter effect on the tremor. This is similar to the co-contractions of healthy subjects to stabilize their upper limbs.

## Discussion

We have proposed algorithms that constitute a first step toward a more intelligent neuroprosthesis for tremor suppression. The algorithms are based on orientation data and respectively estimate sensor location and tremor. Relative sensor location was detected, without any a priori information, with an accuracy of 94.12% for the 4 MIMU configuration, and 100% for the 3 MIMU configurations. We were further able to accurately estimate tremor based on orientation data, with a precision comparable to that of state of the art methods. Using orientation data permits us to identify sensor location, estimate tremor and derive context information from the same dataset, thus reducing bandwidth requirements.

Previous work on detecting sensor location focused on absolute location on the body. However, in many applications sensors are placed in a chain or always on the same site(s). This is particularly the case when biomechanical variables are of interest (e.g., NP control, tele-rehabilitation, motion analysis). In such setups we can deduce absolute position of each sensor from their relative position in the chain. We have therefore opted to determine relative sensor location. The benefit of relative vs. absolute sensor location is that it drastically simplifies the classification and classifier.

Four sensors were used in our study, as is the case in the work presented by Kunze et al. (2005); Kunze and Lukowicz (2007). Several studies have detected more sensors, as many as 17 were identified by Weenk et al. (2013). However, our method is designed with the neuroprosthesis presented in Gallego et al. (2011) in mind, and therefore focuses on one limb consisting of 3 segments. In addition, this is the first attempt to identify various sensors placed on the same segment. The presented method can easily be modified to have less/more sensors or segments, as shown in the different configurations adopted in the present work. This is also supported by previous work on healthy subjects; where the trunk was added as a fourth segment (Lambrecht and Pons, 2014).

To our knowledge this is only the second study looking at identifying sensor location that does not rely on walking data. Kunze et al. have previously published a classifier that was able to determine the location of 4 sensors on specific locations spread across the body from arbitrary movement data. They reported a 82% accuracy on 6 min windows (Kunze and Lukowicz, 2007). We judged that for applications in health and telemedicine this window was too long and the accuracy too low. One of our goals is to facilitate the use of wearable sensors by patients, to make them more user-friendly. Our method was tested on 30 s trials, with actual movement ranging between 15 and 18 s. We are hopeful that this window can be further reduced to incorporate only one movement cycle, without a significant decrease in performance. Although we have only included one task, finger-to-nose test, we believe that our method will perform equally well on related upper limb tasks. The finger-to-nose task shares it dominant kinematic pattern with a variety of ADLs such as eating, drinking, combing your hair, putting on glasses, and answering a phone. Furthermore, no training was needed in the presented algorithm thus there is no indication as to why it should be limited to the finger-to-nose task. Any task that involves motion of the major joints and that triggers kinetic tremor is expected to perform equally well on an essential tremor population.

In recent work by Weenk et al. (2013) an attempt has been made to investigate the sensitivity of location of the sensor on the segment. Previous work has exclusively relied on accelerometer data but Weenk et al. were the first to use gyroscopes as an additional sensor. A slight drop in performance was reported but they still achieved a 97.2% accuracy. In our work we only rely on orientation data. Our algorithm only uses gyroscope and accelerometer data indirectly, as it is based on orientation data. This is the first time orientation data has been used for sensor classification. To further assess the influence of sensor location we included two configurations with 3 MIMUs (i.e., one MIMU per body segment), where the sensor of the forearm was placed distal or proximal. No difference in accuracy was observed. Given the results from both configurations using 3 MIMUs and the fact that none of the features relies on movement to occur about specific axes, we conclude that our method is location and orientation invariant. The features chosen display a high redundancy amongst each other. Future work to identify informative yet complementary features could further increase the precision of the method presented. The current features are individually very discriminative and a combination of features was thus not needed, especially given the high redundancy among them. We did however place the sensors on ideal locations to enable extraction of tremor characteristics. Placing the sensors on different locations and/or orientation would not affect the location identification. The tremor estimate would require a calibration procedure to align the sensor frame to the body-segment frame. Soft tissue artifacts might further filter part of the signal and/or introduce noise through wobbling masses.

We estimated tremor based on orientation data following the protocol presented in Gallego et al. (2010) for gyroscope data. We compared our results to those obtained using both an online estimation method and an offline reference method. Our results show, for the first time, that it is possible to accurately track tremor collecting only orientation data. The orientation based method does appear to have more difficulties adapting quickly to changing patterns. This observation was supported by the overall slightly larger values for the first component of the KTE (i.e., the mean absolute estimation error), the figure of merit used to compare the performance of the tremor tracking methods. Difference in performance relative to gyroscopes was particularly noticeable upon changes in velocity. This is most likely due to the nature of the EKF and the parameters defining it. Although we did not have access to the exact parameter values, we believe that altering the fusion filter or the filter parameters can improve the performance of the presented method. As is, the EKF is set to perform well for normal human motion, situated below 2 Hz in the frequency spectrum. Higher sensitivity to changes up to 8–10 Hz and a faster response time will most likely preserve the tremulous movement better and thus result in a better estimate. Further work, with customizable fusion algorithms, is needed to confirm this hypothesis.

The ability to track tremor with orientation data simplifies demands for bandwidth and processing power when incorporated in monitoring applications. It constitutes a significant step toward a more intelligent neuroprosthesis for tremor suppression and opens the door for long-term continuous tremor monitoring with context awareness.

Our future work will be directed toward adding a task-identifier based on joint angles and joint angle related features to these algorithms; validating the sensor location algorithm on other types of tremor patients and different pathologies, as well as use the presented work to investigate context and evolution of tremor occurrence.

## Conclusion

The work described in this paper constitutes the first steps toward a more user-friendly and context-aware neuroprosthesis for tremor suppression and monitoring. We predict that this methodology will enable the monitoring of tremor with context awareness and will facilitate the use of wearable sensors in tele-health and tele-medicine applications.

We have introduced a method to automatically identify relative sensor location. This is the first location detection algorithm based on orientation data, the first that only requires upper limb movement and does not need any training, and only the second to be tested on a patient population.

We furthermore introduced an algorithm to track tremor using orientation data. As a direct application we will use this in the long-term monitoring of tremor characteristics and context.

## Author contributions

Stefan Lambrecht designed the study, developed the algorithms, performed the literature review, and drafted the manuscript. Juan Alvaro Gallego and Eduardo Rocon collected the data and revised the manuscript. Eduardo Rocon and Jose Luis Pons supervised this research. All the authors have read and approved the final manuscript.

### Conflict of interest statement

The authors declare that the research was conducted in the absence of any commercial or financial relationships that could be construed as a potential conflict of interest.
